# Star intrapreneurs:characteristics of Indonesian corporate entrepreneurs

**DOI:** 10.1016/j.heliyon.2022.e12700

**Published:** 2022-12-30

**Authors:** Denny Bernardus, Jimmy Ellya Kurniawan, Fulgentius Danardana Murwani, Jony Eko Yulianto

**Affiliations:** a Universitas Ciputra Surabaya, Indonesia; b Massey University, Auckland, New Zealand; c Universitas Negeri Malang, Indonesia

**Keywords:** Intrapreneur, Corporate entrepreneur, Indonesia

## Abstract

Global competition has been driving many companies globally, including in Indonesia, to develop intrapreneurship within their organizations. The current study offers the concept of star intrapreneurs to examine the intrapreneur character scale which operates at the individual level within the cultural context of Indonesia. The concept of intrapreneurship in this study consisted of two dimensions: the person and the interaction. The dimension of person has three sub-dimensions: pattern of constructive thinking, pattern of positive attitude, and pattern of productive action. Meanwhile, the dimension of interaction also consisted of three subdimensions: friend in thinking, interlocutor, and partner in action. Visually, the six sub-dimensions are depicted in the form of a star with six corner points. Confirmatory factors analysis was used. This study has proven that the two dimensions and the six sub-dimensions of star intrapreneurs are acceptable and correlate with individual work performance as an external validator.

## 1 Introduction

Currently, there are increasing numbers of company developing corporate entrepreneurship. The ratio of entrepreneurial firms in the United States increased by more than 1.5 times from 1975 to 2010 [1]. In the company, employees are required to improve their entrepreneurial skills to increase their competitiveness and develop their future careers [2]. The increasingly high global competition and demands for efficiency also increase the need for entrepreneurship within companies in order to survive and generate profits by developing a competitive advantage [3].

The term corporate entrepreneurship is often used to describe entrepreneurial behavior in already operating large and medium scale organizations. Corporate entrepreneurship is also often referred to as *intrapreneurship* [4]. Historically, Gifford Pinchot popularized the term intrapreneurship in 1978, which stands for intra-corporate entrepreneurship, as the process of taking responsibility for developing innovations in various ways within an organization and demonstrating how to turn an idea into real profit. Furthermore, Pinchot initiated *The Intrapreneur's 10 Commandments*, which provides tips to intrapreneurs. The intrapreneur tips often encourage employees to take the risk of acting outside of company procedures, promoting being prepared to apologize if caught rather than experiencing difficulties gaining permission, and even promote being prepared to be fired [5,6].

Although the term corporate entrepreneurship is often used interchangeably with intrapreneurship, [7] explained the difference between corporate entrepreneurship and intrapreneurship. Corporate entrepreneurship refers to entrepreneurial activities at the organizational level, while intrapreneurship refers to entrepreneurial activities at the individual or employee level within an organization. Over the past few decades, entrepreneurial characteristics, also known as entrepreneurial orientation, have begun to be applied at the individual level in employees, university students, and even high school students [[8], [9], [10], [11], [12]]. However, entrepreneurial concepts and characteristics in previous studies indicate more personal achievement competences than interaction competences, as stated in Table 1 and 2 [10,11,[13], [14], [15], [16], [17], [18], [19], [20], [21], [22], [23]].

**Table 1:** Entrepreneurial concepts and characteristics.

Author	Concept	Level	Characteristics
Bulgerman [13]	Corporate entrepreneurship is the process of diversifying a company through the use of various resources that were not previously relevant to create new areas of competence in order to take an opportunity.	Organizational	1. Autonomous Strategic Behavior (Strategic Context) 2. Induced Strategic Behavior (Structural Context)
Miller [14]	An entrepreneurial firm is one that engages in product-market innovation, undertakes somewhat risky ventures, and is first to come up with ‘proactive’ innovations, beating competitors to the punch.	Organizational	1. Innovation 2. Proactiveness 3. Risk-taking
Covin & Slevin [15]	Entrepreneurial firms are those in which the top managers have entrepreneurial management styles, as evidenced by the firms’ strategic decisions and operating management philosophies.	Organizational	1. Risk-taking 2. Innovation 3. Proactiveness
Guth & Ginsberg [16]	Corporate entrepreneurship activities include new venture creation in existing companies and organizational transformation using renewal strategies	Organizational	1. Innovation/Venturing within Established Corporations 2. Strategic Renewal of Established Corporations
Zahra [17]	Corporate entrepreneurship as both formal and informal activities that aim to develop new businesses within existing companies through product and process innovations, and market development.	Organizational	1. Product Innovation 2. Risk-Taking 3. Proactiveness
Carrier [18]	Intrapreneurial characteristics are not the exclusive property of employees of large firms. Intrapreneurs can be first-class allies for owner-managers of growing small businesses.	Organizational	1. Intrinsic personality - related motivations 2. Extrinsic personality - related motivations 3. Motivations related to past experience and future career objectives 4. Motivations related to the organizational context
Lumpkin & Dess [19]	Entrepreneurial Orientation refers to the processes, practices, and decision-making activities that lead to new entry” as characterized by one, or more of the following dimensions: “a propensity to act autonomously, a willingness to innovate and take-risks, and a tendency to be aggressive toward competitors and proactive relative to marketplace opportunities	Organizational	1. Autonomy 2. Innovativeness 3. Risk taking 4. Proactivenss 5. Competitive Aggressiveness
Antoncic & Hisrich [20]	Intrapreneurship is viewed as being beneficial for revitalization and performance of corporations, as well as for small and medium-sized enterprises.	Organizational	1. New Business Venturing 2. Innovativeness 3. Self-renewal 4. Proactiveness
Sayeed & Gadzar [21]	Intrapreneurship is composites of seven dimensions, namely, imagination, intuition, authority, will, sociability, energy and flexibility.	Individual	1. Imagination (Innovator) 2. Intuition (New Designer/Enabler) 3. Authority (Leader) 4. Will (Entrepreneur) 5. Sociability (Animateur) 6. Energy (Advanturer) 7. Flexibility (Change Agent)
Walcott & Lippitz [22]	Corporate entrepreneurship is the process by which teams within an established company conceive, foster, launch and manage a new business that distinct from but leverages the parent firm's assets, market position and capabilities. There are three important aspects of corporate entrepreneurship: strategic updates related to organizational revitalization (strategic and structural changes), innovation related to efforts to introduce new products to the market, and corporate venturing, which is related to entrepreneurial efforts to develop new corporate organizations within a company.	Organizational	1. Strategic updates related to organizational revitalization (strategic and structural changes) 2. Innovation related to efforts to introduce new products to the market 3. Corporate venturing, which is related to entrepreneurial efforts to develop new corporate organizations within a company.
Bolton & Lane [10]	Entrepreneurs’ willingness to take on risks and be proactive in leading their organisation can certainly be important behaviours that a person may take on in other pursuits of life.	Individual	1. Innovativeness 2. Risk-taking 3. Proactiveness
Kurniawan et al. [11]	Entrepreneurial orientation is behavioral perception about innovativeness, risky proactiveness and competitiveness.	Individual	1. Innovativeness 2. Risky proactiveness 3. Competitiveness
[23]	Intrapreneurship is crucial for employees and managers, who participate in the team to transform the organization to be capable of applying new business practices through innovation and process/technology.	Individual	1. Product/service 2. Innovation 3. Process/technology and new businesses

**Table 2:** Fit model.

	Saturated Model	Estimated Model
SRMR	0.060	0.068
d_ULS	0.101	0.130
d_G	108.412	111.547
Chi-Square	2.529.089	2.573.083
NFI	0.208	0.194

Table 1 shows that prior studies generally have discovered entrepreneurial characters at the organizational level. Relatedly, there are not many studies that examine entrepreneurial characters at the individual level. The most studies of entrepreneurial characteristics have focused more to describe personal achievement and other individual competencies, such as being innovative, risk-taking, proactive, autonomy, competitive, and so forth. This study is useful particularly to understand the trait of an intrapreneur as an individual. However, intrapreneurs do not work in a social vacuum. People in organizations need to work collaboratively to be able to co-create innovations and test new ideas. As such, we would argue that central in intrapreneurship is also the relational aspects, such as inter-personal interactions. However, such relationality is limited and warrants further research, which we directly address in this paper.

Conceptually, Narasimhan [24] defines intrapreneurship as the process in which individuals within an organization introduce innovations that are considered to be valuable to the managers using processes that are appropriate and acceptable to the dominant norms within the organization (organizational understanding). For example, some company managements are more focused on and appreciative of the commercialization value of new technologies, while other companies are more focused on and place greater value on cutting costs. Intrapreneurs must adhere to the values held by the company's management and reframe innovations to fit these values in order that they do not conflict with relevant company procedures. Thus, innovative ideas are more likely to be realized [24]. Thus, an intrapreneur must also have interaction competencies to better understand organizational norms in order to realize their innovations.

The need for intrapreneurial expertise in adapting to corporate norms is even more crucial for intrapreneurs working in Indonesia, a country with high power distance and collectivism culture. Organizations or workplaces in countries with a high power distance are characterized by centralization, greater reliance on superiors and formal regulations within the workplace. They consider leaders as “the good fathers” to their subordinates, and the subordinates tend to follow the style of their superiors [25]. Employees working in high power distance cultures are expected to be liked and trusted by their superiors, especially among employees who expect a high resource dependence relationship with their superiors [26]. Meanwhile, organizations or workplaces in collectivist countries possess the characteristic of placing great importance on the opinions and joint decision of their in-group. The management is run by the in-group of the top leader, meaning that employees who wish to be treated better must approach and adjust to the norms of the management team in-group [25].

Intrapreneurs who work in cultures of high power distance and collectivism, such as in Indonesia, need leaders in the top management as the persons who support them to realize their innovative ideas and self-development. Previously, scholars have noted that intrapreneurs often have a motive to develop themselves within a corporation, are oriented toward serving themselves, customers, and sponsors, and conduct transactional relations within their corporate hierarchies [27]. As suggested by Narasimhan [24]; intrapreneurs should introduce their innovations in a way that conforms with the corporation's norms. Adjustment to the corporation's norm requires interaction competences that, however, are rarely studied in previous intrapreneurship studies.

As a direct response to this research gap, the current study aims to examine an intrapreneur character scale at the individual level in Indonesian context. The scale is referred to as star intrapreneurs, and the dimensions not only emphasize the individual's characteristics as an entrepreneurial figure or person, but also their characteristics in terms of interacting in accordance with the norms of their workplace [28].

### 1.1 Star intrapreneurs

**Fig. 1:**
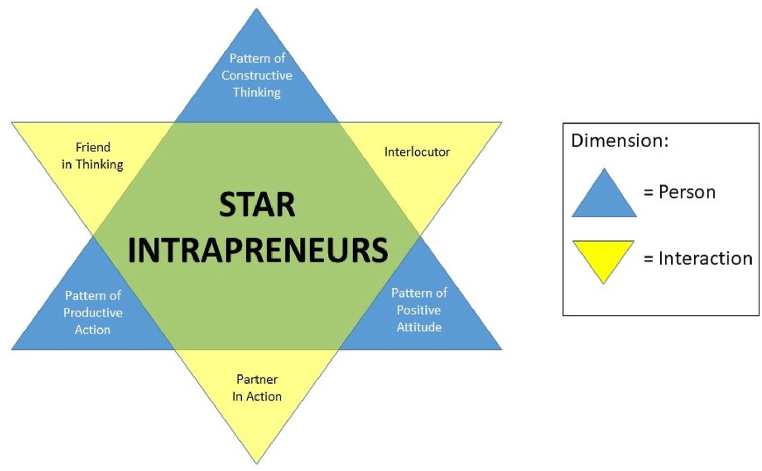
Star intrapreneurs.

The concept of star intrapreneurs arose from the dynamics of internalization, interaction, and actualization (DIIA) within the framework of corporate entrepreneurship. The characteristics of an intrapreneur consist of two dimensions: the person and the interaction. The dimension of person consists of three sub-dimensions: pattern of constructive thinking, pattern of positive attitude, and pattern of productive action. Meanwhile, the dimension of interaction also consists of three sub-dimensions: friend in thinking, interlocutor, and partner in action. The two dimensions and each of their three sub-dimensions are depicted in the form of two inter-related triangles that form a “star”, resulting in the concept being termed star intrapreneurs, as seen in Fig. 1 [28].

The dimension of person refers to the entrepreneurial characteristics possessed by intrapreneurs. The pattern of constructive thinking subdimension is indicated by individuals who maintain a positive attitude when facing challenges, and who rarely complain, stay focused and diligent when facing challenges, and are willing to pay the price to achieve long-term goals [29–30]. The pattern of positive attitude subdimension is indicated by individuals who never give up at work, are open-minded, are caring and friendly to their colleagues and subordinates, and who like to explore and seek out new opportunities [31–32]. The pattern of productive action subdimension is indicated by individuals who immediately complete tasks without procrastinating (including unpleasant tasks), handle responsibilities enthusiastically and passionately, and plan and manage their time well [33–34].

The dimension of interaction refers to the characteristics required by an individual intrapreneur in interacting with the prevailing norms within their workplace. The friend in thinking subdimension indicates an individual who voluntarily helps their colleagues and subordinates who are experiencing difficulties at work, and who actively provides information related to their area of expertise to colleagues or departments that need it [35,36]. The interlocutor subdimension indicates an individual who values, responds positively to, and is liked by their colleagues and subordinates, and who is open to feedback from their superiors, colleagues, and subordinates to improve their performance [37,38]. The partner in action subdimension indicates an individual who believes in and supports the overarching policies at work, who can be relied upon to plan, implement, and follow-up their work, and who supports the organizations goals and is committed to achieving them with all of their might [[39], [40], [41], [42]].

### 1.2 Intrapreneur and individual work performance

Individual Work Performance is defined as behaviors or actions that are in accordance with or relevant to an organization's goals [43]. Many studies have proven that intrapreneurship has an effect on individual work performance. Scholars have investigated that intrapreneurship can increase the performance, market share, sales, and size of medium-sized companies [44]. Intrapreneurial behaviour also has a positive impact on job performance engineers and managers working in multinational companies [45]. A conceptual research framework assumed intrapreneurial behavior to be a mediator in the effects of psychological safety and psychological empowerment on middle manager individual performance [46]. One study also proved that intrapreneurship mediates the relationship between psychological ownership and teachers’ role performance [47]. Intrapreneurial behavior has also been proven to mediate the relationship between psychological safety and individual performance of production/operations managers of medium enterprises (MEs) [46]. The body of knowledge above demonstrated that intrapreneurship has a strong correlation with work performance, including at an individual level. This means that individual work performance should be an appropriate variable to test the external validity of the Star Intrapreneur scale.

According to Koopmans et al. [43] there are three dimensions of individual work performance: task performance, contextual performance and counterproductive work behavior. The task performance dimension is an individual's ability to or expertise in completing substantive tasks and their main job responsibilities. The contextual performance dimension is individual's behaviors or attitudes that support the organizational, social, and psychological environment in regard to the main functions of their job. Meanwhile, the counter-productive work behavior dimension, which refers to individual behavior that is detrimental to the organization's welfare, will not be used to test the external validity of this study as it is contrary to the characteristics of an intrapreneur.

## 2 Method

The current study adopted a quantitative approach using a correlational method implemented among a group of companies that own businesses in the housing sector (real estate, malls, theme parks) and the social sector, namely education (play groups, kindergarten, elementary school, junior high school, and senior high school), that are spread across 12 major cities in Indonesia, which have central role in contributing economic development in the country.

The research was conducted in two studies. The first study involved 76 participants, which consisted of 25 men and 51 women, age range between 24 and 53 years, with 3 participants having completed senior high school, 6 participants having completed a diploma, 58 participants having completed a bachelor degree, and 9 participants having completed a post-graduate degree. Their periods of service ranged between 1 and 19 years, with 53 participants working in the education sector and 23 participants working in the housing sector, 43 participants occupied managerial positions and 33 participants were non-managerial staff members.

The second study involved 232 participants consisting of 118 men and 114 women, between the ages of 22 and 56 years, with 3 participants having completed junior high school, 48 having completed senior high school, 23 participants having completed a diploma, 144 participants having completed a bachelor degree, and 14 having completed a post-graduate degree. Their periods of service ranged between 1 and 27 years, with 73 individuals working in the education sector and 159 participants working in the housing sector, 95 participants occupied managerial positions and 137 participants were non-managerial staff members.

Prior to the development of the scale, focus group discussions were held with psychology expert, lead statisticians, and HCM leaders from the company, in both the education and housing sectors. Based on the results of the focus group discussions, the star intrapreneur scale was developed in Indonesian language, consisting of person and interaction dimensions. Participants were asked to respond to each item using a 5 point Likert scale (1 = very inappropriate, 5 = very appropriate). All participants provided written informed consent prior to responding to the survey.

The dimension of person, which refers to the entrepreneurial characteristics possessed by individual intrapreneurs, consists of 55 items arranged based on indicators that reflect patterns of constructive thinking, patterns of positive attitude, and patterns of productive action. The pattern of constructive thinking items were modified from the Constructive Thinking Inventory - Behavior Coping items from Creative Problem Solving developed by Epstein [30]. This is including the item of “I am more of the action-taking type than the complaining type”. The Focus and Analytical items developed by Constantin et al. [48]. For example, “Once I have decided to do something, I will pursue it without giving up until I reach my goal”. The long term orientation items developed by Bearden et al. [29]. The example of the item is, “I have a long-term plan”. The pattern of positive thinking items were modified from the persistence or grit items developed by Duckworth et al. [49]; including “I have reached a goal that required years of dedication”, the pleasant personality or socio-emotional communication items developed by Cegala et al. [31]. For example, “I have a warm and positive attitude toward others”, and the flexible and open minded and curiosity items developed by Sulistiani et al. [32]; including “I wish to know about new opportunities”. The pattern of productive action items were modified from the non-procrastination items developed by Metin et al. [33]; including “I immediately start work that I am required to do”, the enthusiasm and responsibility or vigorous items developed by Schaufeli & Bakker [50]; including “At my workplace, I feel full of energy”, and the time management items developed by Xue & Sun [34]; including “I make a to-do list every day”.

The dimension of interaction, which refers to the characteristics required by individual intrapreneurs in interacting with the prevailing norms in their organizations, consists of 38 items arranged based on indicators that reflect friend in thinking, interlocutor, and partner in action aspects. The friend in thinking items were modified from the helpful or altruism items developed by Podsakoff and colleagues (1990), including “I help other people who are experiencing a heavy workload”, and the resourceful in terms of topic or information dissemination items developed by Schlosser & McNaughton [36]; including “I inform the appropriate department when I learn of something important regarding my area of expertise”. The interlocutor items were modified from the respect to others and respect to close relationships items developed by Hendrick & Hendrick [37]; including “I value my co-workers”, and the easily accept feedback and criticism and utility items developed by Linderbaum & Levy [38]; including “Feedback contributes to my success at work”. The partner in action items were modified from the concomitant and in-arms and submission to authority items developed by Dunwoody & Funke [39]; including “I believe what my boss says”, the reliable and trustworthy and conscientiousness items developed by Ramdhani [41]; including “I am a reliable worker”, and the commitment to a common goal or goal commitment items developed by Seijts & Latham [42]; including “I am extremely committed to pursuing the goals of the organization”.

Confirmatory factor analysis (CFA) was used to analyze the data to obtain construct validity in proving the star intrapreneur scale model. Item parceling [51] was used in CFA. Star intrapreneurs was measured by two dimensions, namely person and interaction. Person was measured by three indicators (manifest variables), namely pattern of constructive thinking, pattern of positive attitude, and pattern of productive action. Interaction was measured by three indicators, namely friend in thinking, interlocutor, and partner in action.

External validity was tested by correlating the Star Intrapreneur scale with the Individual Work Performance measurement modified by Koopmans et al. [43]. The Individual Work Performance scale consists of five items for the dimension of task performance, including “I create a work plan so that my work is completed on time”, and eight items for the contextual performance dimension, including “I take on extra responsibilities”.

## 3 Results

The results of the first study involving 76 employees revealed that the three person indicators: pattern of positive attitude, pattern of productive action, and pattern of constructive thinking, are significant indicators for the person dimension. Outer loading for pattern of positive attitude = 0.942 with a path coefficient = 71.064 (p = 0.000; p < 0.05). Outer loading for pattern of productive action = 0.916 with a path coefficient = 44.904 (p = 0.000; p < 0.05). Meanwhile, outer loading for pattern of constructive thinking = 0.919 with a path coefficient = 32.965 (p = 0.000; p < 0.05). The R square value for the person dimension was 0.930, meaning that the three indicators account for 93% of the total value of the person dimension. The person dimension had an Average Variance Extracted (AVE) = 0.857; Composite Reliability = 0.947 and Cronbach Alpha = 0.917.

In the first study, the three interaction indicators: friend in thinking, interlocutor, and partner in action, were also significant indicators of the interaction dimension. Outer loading for friend in thinking = 0.889 with a path coefficient = 30.154 (p = 0.000; p < 0.05). Outer loading for interlocutor = 0.863 with a path coefficient = 25.167 (p = 0.000; p < 0.05). Meanwhile, outer loading for partner in action = 0.931 with a path coefficient = 74.670 (p = 0.000; p < 0.05). The R square value for the interaction dimension was 0.928, meaning that the three indicators account for 92.8% of the total value of the interaction dimension. The interaction dimension had an Average Variance Extracted (AVE) = 0.801; Composite Reliability = 0.923 and Cronbach Alpha = 0.875.

According to the results of the first study, the path coefficient for the person dimension = 0.964. Likewise, the path coefficient for the interaction dimension = 0.964. The F square value for the person dimension of the star intrapreneur scale was 13.306 and the F square value for the interaction dimension in the star intrapreneur scale was 12.984. The model is fit with the following criteria on Table 1.

The results of the second study involving 232 employees also show that the three person indicators: pattern of positive thinking, pattern of productive action, and pattern of constructive thinking, are significant indicators of the person dimension. The Standardized Regression Weight (outer loading) for pattern of positive attitude = 0.914 with an estimated regression weight = 0.914 (p = 0.000; p < 0.05). The Standardized Regression Weight (outer loading) for pattern of productive action = 0.962 with an estimated regression weight = 1.000 (p = 0.000; p < 0.05). The Standardized Regression Weight (outer loading) for pattern of constructive thinking = 0.909 with an estimated regression weight = 0.518 (p = 0.000; p < 0.05).

In the second study, the three interaction indicators: interlocutor, partner in action, and friend in thinking, were also significant indicators of the interaction dimension. The Standardized Regression Weight (outer loading) for partner in action = 0.916 with an estimated regression weight = 1.000 (p = 0.000; p < 0.05). The Standardized Regression Weight (outer loading) for interlocutor = 0.829 with an estimated regression weight = 0.479 (p = 0.000; p < 0.05). The Standardized Regression Weight (outer loading) for friend in thinking = 0.864 with an estimated regression weight = 0.564 (p = 0.000; p < 0.05).

The model from the results of the second study is good fit. Although chi-square = 35.468 and p-value = 0.000, indicating that the model is not fit, the model still good fit with the other criteria, with GFI = 0.950; CFI = 0.982 and TLI = 0.966. Meanwhile, RMSEA = 0.122 or almost fit as it is approaching 0.08. The accepted star intrapreneur model is presented in the following figure.

**Fig. 2:**
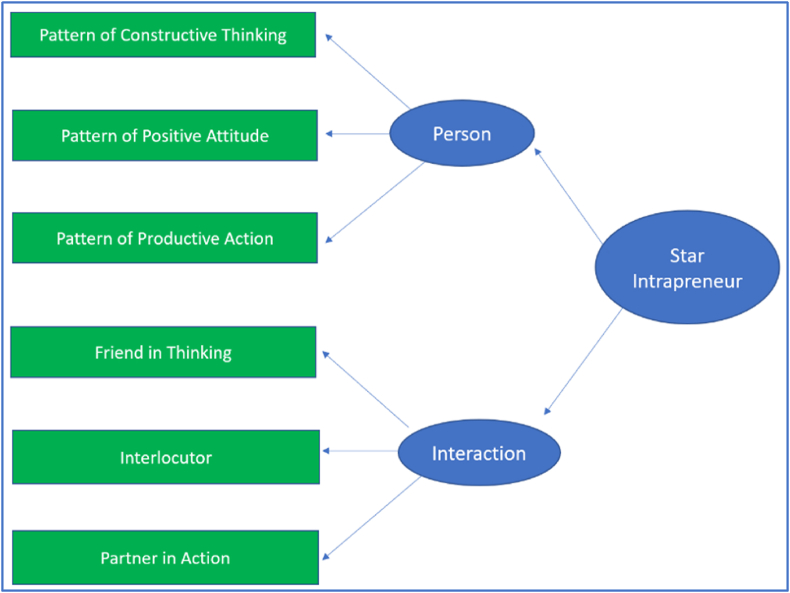
Star intrapreneur model.

Based on Fig. 2, this study proves that all sub-dimensions of person have a valid construct, including all sub-dimensions of interaction. This figure also proves that the dimensions of person and interaction are valid constructs for intrapreneurs in this population in Indonesia.

The external validity of the star intrapreneur scale was tested by examining the correlation with individual work performance, consisting of the dimensions of task performance and contextual performance. The individual work performance scale reliability test results revealed that the reliability of Cronbach Alpha was 0.847 for the task performance dimension and 0.892 for the contextual performance dimension. The assumption test and the normality test on the distribution of individual work performance data showed that Shapiro-Wilk = 0.953 and p < 0.001, meaning that the data distribution is not normal, and, therefore, the external validity was tested using a non-parametric approach; the Spearman Rho correlation. The results of the external validity correlation between the dimensions and variables of the star intrapreneur scale with individual work performance are presented in the following on Table 3.

**Table 3:** Correlation of external validity.

Variable, Dimension and Indicator	Spearman Rho's Correlation		
	Task Performance	Contextual Performance	Individual Work Performance
Star Intrapreneur	0,794 **	0,853 **	0,894 **
Person	0,790 **	0,841 **	0,882 **
Pattern of Constructive Thinking	0,721 **	0,804 **	0,823 **
Pattern of Positive Attitude	0,699 **	0,768 **	0,794 **
Pattern of Productive Action	0,779 **	0,789 **	0,845 **
Interaction	0,727 **	0,807 **	0,835 **
Friend in Thinking	0,633 **	0,754 **	0,762 **
Interlocutor	0,589 **	0,631 **	0,664 **
Partner in Action	0,704 **	0,765 **	0,794 **

**p < 0,001.

Based on Table 3, this study proves that this model is also externally valid where intrapreneur star is positively correlated with individual work performance and each of its dimensions, namely task performance and contextual performance. Both of star intrapreneur dimensions, namely person and interaction with their respective sub-dimensions are also positively correlated with individual work performance and each of its dimensions.

## 4 Discussion

The star intrapreneur models for both studies are proven good fit. In accordance with the assumptions of Wahjudono [28]; the current research proves that the star intrapreneur scale, which describes the characteristics of intrapreneurs in Indonesia, is divided into two dimensions: person and interaction. The person dimension was proven to have three indicators: pattern of constructive thinking, pattern of positive attitude, and pattern of productive action. Meanwhile, the interaction dimension was also proven to have three indicators: friend in thinking, interlocutor, and partner in action.

The intrapreneurship model, which is not solely built on achievement person competences, but is also built on interaction competences, is in line with the context in Indonesia, which has a culture of high power distance and collectivism [25]. The culture in Indonesia demands that intrapreneurs need to adapt to the corporation's norm to realize their innovations [24], so it is not enough to rely solely on achievement of person competences, but requires interaction competences.

The external validity test also proved that there is a positive relationship between the variables, dimensions, and indicators of the star intrapreneur scale and individual work performance. The star intrapreneur variables, dimensions, and indicators also have a positive relationship with each of the dimensions: task performance and contextual performance. As previously described by Koopmans et al. [43]; the task performance dimension is associated to an individual's ability and expertise in completing substantive tasks and their main work responsibilities. Meanwhile, the contextual performance dimension refers to an individual's behaviors or attitudes that support the organizational, social, and psychological environment in regard to the main functions of their job.

Pattern of constructive thinking positively correlates with task and contextual performance. Positive energy and focus on achieving long-term goals are the skills and attitude required by individual intrapreneurs to produce good work performance. Achieving personal work goals and objectives is one competency dimension required to work in the era of Industry 4.0 [52]. Thus, patterns of constructive thinking may support individual work performance.

Pattern of positive attitude positively correlates with task and contextual performance. Persistence and being open to exploring new opportunities are the skills and attitudes required by individual intrapreneurs to produce good work performance. Opportunity exploration, which is a form of entrepreneurial and commercial thinking, is one competency dimension required to work in the era of Industry 4.0 [52]. Thus, patterns of positive attitude may support individual work performance.

Pattern of productive action positively correlates with task and contextual performance. The ability to manage one's time and complete responsibilities without procrastinating is one of the skills and attitudes required by individual intrapreneurs to produce good work performance. Completing tasks on time is a form of task accomplishment and management of work performance [43]. Thus, patterns of productive action may support individual work performance.

Friend in thinking positively correlates with task and contextual performance. Voluntarily assisting and actively providing information to colleagues and superiors is one of the skills and attitudes required by individual intrapreneurs to produce good work performance. Helping to voluntarily provide information to colleagues and superiors is one example of being able to work in a team and the ability to transfer knowledge to others, both of which are important competencies in smart factories [53]. Thus, being a friend in thinking may support individual work performance.

Interlocutor positively correlates with task and contextual performance. Valuing and being open to feedback from superiors and colleagues, and responding positively, are some examples of the skills and attitudes required by individual intrapreneurs to produce good work performance. Being open to feedback can influence one's job performance [54]. Thus, being an interlocutor may support individual work performance.

Partner in action positively correlates with task and contextual performance. Individual intrapreneurs who support policies, are committed, and can be relied on by their superiors/the organization are a manifestation of the skills and attitudes required to produce good work performance. Commitment and the ability to be relied upon be the organization are forms of organizational citizenship behavior that influence employee performance [55]. Thus, being a partner in action may support individual work performance.

To conclude, this study offers an empirical evidence that the two dimensions and the six sub-dimensions of star intrapreneurs are proven acceptable and correlate with individual work performance as an external validator. The results of the current study contribute to the existing scholarship by providing an intrapreneurial characteristic scale at the individual level that is more suitable to the cultural and organizational context of Indonesia. The practical implication of the results of this study is the application of the star intrapreneur scale in the selection and promotion process, as well as training, for companies in Indonesia that require a lot of intrapreneurs. This research has a limitation, in the sense that it was only conducted with the involvement of one group of companies. It is recommended that further studies need to test the star intrapreneur scale on companies from different sectors in Indonesia that may have different organizational cultures to the group of companies examined in the current study.
